# E-Cigarette as a Harm Reduction Approach among Tobacco Smoking Khat Chewers: A Promising Bullet of Multiple Gains

**DOI:** 10.3390/ijerph13020240

**Published:** 2016-02-19

**Authors:** Saba Kassim, Konstantinos E. Farsalinos

**Affiliations:** 1 College of Dentistry, Taibah University, Al-Madinah Al-Munawwarah 43353, Saudi Arabia; 2 Onassis Cardiac Surgery Center, Sygrou 356, Kallithea 17654, Greece; kfarsalinos@gmail.com

**Keywords:** khat, tobacco smoking, e-cigarettes, nicotine

## Abstract

Khat chewing/use, a green leaf with amphetamine-like effects is socially integrated in the Middle East and Africa. Khat chewing is often associated with tobacco smoking and occurs in closed places, such as a family home setting where the smoke-free laws cannot be implemented. Tobacco cigarette smoking among khat chewers is a significant concern, but there is also second-hand exposure to smoke at home or in places where khat users gather. Evidence suggests that e-cigarettes represent a significantly less harmful form of nicotine intake. Evaluating the effects of e-cigarettes among khat chewers could be important in understanding the impact of e-cigarettes as a harm reduction approach, with the potential to reduce the health risk associated with smoking.

## 1. Introduction

The chewing/use of the amphetamine–like khat leaf from Catha *edulis* is widespread and socially ingrained in the Middle East and East Africa and their diaspora communities world-wide. Khat chewing has become a national and international public health concern [1] and has recently gained popularity globally [2]. Khat is very often used with tobacco smoking [3]. Khat chewers are either daily tobacco smokers or smoke tobacco when chewing khat [3]. The use of tobacco by khat chewers represents a clear public health concern [4]. There is a significant prevalence of tobacco smoking among adult and school children khat chewers [4] with gender difference in mode of tobacco use: women often smoke waterpipe tobacco whereas men often smoke tobacco cigarettes [5]. An alarming elevation in the level of tobacco dependence associated with higher khat dependence, and very high levels of carbon monoxide among smoking khat chewers have been observed [3]. The estimated of tobacco use among khat users *versus* smoker none khat users was found higher [4] although the difference in smoking dependence between khat users and non-users has not been established.

The reason that khat users smoke tobacco is mainly to enhance the stimulant effects of khat [3], indicating some interaction between nicotine/tobacco and the chemicals present in khat that awaits identification [3]. The session of khat-use often occurs in closed places, such as a family home setting or meeting places for immigrants [6,7] where the smoke-free laws cannot be implemented. Thus, besides the risks associated with smoking among khat chewers, there is also second and third-hand (environmental) exposure to the toxicants present in cigarette/waterpipe smoke.

Khat chewing is an integral part of different social aspects, and attempts to control its use failed in khat-originating countries [8]. However, efforts should be made to reduce the adverse effects of smoking in khat chewers. Although many khat users reported they had thought about and had attempted to quit khat and tobacco use [5], there is no evidence on the effects of using approved smoking cessation methods in this population. Moreover, access to and willingness to visit smoking cessation clinics is expected to be limited, considering that most khat users are immigrants or living in low income countries (e.g., Yemen) where implementation of tobacco control and availability of tobacco cessation services are scarce. One of the methods of promoting smoking reduction or cessation would be by switching from inhalation of combustible products to a non-combustible nicotine-delivery product, such as the e-cigarette. The emergence of the e-cigarette as a harm reduction product that could decrease smoking related disease has currently received great attention among regulators and the scientific community. Evidence suggests that, although not absolutely safe [9], it represents a significantly less harmful form of nicotine intake [10,11] and can become an important tool for smoking reduction [12] and cessation [13], even in highly-dependent smokers (based on Fagerström Test for Cigarette Dependence) with very high daily cigarette consumption [14]. Thus, e-cigarettes could be used as a harm-reduction approach, with the main goal of reducing the adverse health effects of smoking in the population of khat users. However, the acceptability and subsequent effects of e-cigarette use has not been tested among tobacco smoking khat chewers. E-cigarettes could be used as an alternative to smoking for this population, due to their ability to deliver nicotine (which is needed to enhance khat effects) as well as substitute the sensory-motor aspects of the smoking behavior. Additionally, their use could potentially reduce second and third-hand exposure to the tobacco cigarette smoke observed in rooms where khat-chewing sessions are performed.

## 2. Future Research Directions

Future research should consider evaluating the use of e-cigarettes in khat chewing sessions as a specific need within the broader general need for research regarding the safety and efficacy of e-cigarettes [15]. Different research questions related to tobacco use along with khat chewing should be tested, including the following:

1. Can e-cigarettes enhance the effect of khat use similarly to tobacco smoking?
2. Can e-cigarettes effectively reduce the need of khat chewers to smoke, leading to smoking reduction or cessation?
3. Can e-cigarettes have an effect in reducing khat use?
4. Will khat chewers who use waterpipe (predominantly women) accept the use of e-cigarette products that resemble waterpipe smoking (there are electronic shisha products with design similar to waterpipe which use e-cigarette liquid and produce aerosol similar to conventional e-cigarettes)?
5. Will partial or complete switching from tobacco smoking to e-cigarette use result in reduced biomarkers of toxins exposure (nitrosamines, polycyclic aromatic hydrocarbons, *etc.*) in khat chewers?

## 3. Conclusions

In khat chewing countries, e-cigarettes could become a promising bullet that might reduce the health risks of smoking khat chewers and the second and third-hand exposure of people living around them. That would be consistent with the Framework Convention on Tobacco Control and Tobacco Treatment (FCTC) goals of treating tobacco dependence and protecting people from second-hand exposure while taking into account national circumstances and priorities [16]. With the current opinion letter we propose the development of a new research agenda, focusing on the efficacy of e-cigarette use on tobacco smoking khat chewers.
